# Direct simulation of hypertensive stress on endothelial cells: a streamlined model of in-vitro-hypertension

**DOI:** 10.3389/fphys.2025.1724932

**Published:** 2026-01-14

**Authors:** Elena Raschi, Caterina Bodio, Chiara Brullo, Gianfranco Parati, Pier Luigi Meroni, Maria Orietta Borghi, Laura Calvillo

**Affiliations:** 1 Immunorheumatology Research Laboratory, IRCCS Istituto Auxologico Italiano, Milan, Italy; 2 Department of Pharmacy, Section of Medicinal Chemistry, University of Genova, Genova, Italy; 3 Department of Cardiology, Istituto Auxologico Italiano IRCCS, Milan, Italy; 4 Department of Medicine and Surgery, University of Milano-Bicocca, Milan, Italy; 5 Dipartimento di Scienze Cliniche e di Comunità, Dipartimento di Eccellenza 2023-2027, University of Milan, Milan, Italy

**Keywords:** human umbilical vein endothelial cells, hypertension, bioreactor, Live-Pa, Angiotensin II, dynamic culture, millifluidic *in-vitro* technology, 3Rs

## Abstract

Hypertension stands as one of the most significant preventable risk factors for cardiovascular disease, which is the leading cause of mortality worldwide. There is a disturbing gap, in preclinical research, between simplified cell culture and complex *in vivo* models. To contribute to bridge this gap, we have developed a simplified but realistic *in vitro* dynamic model of hypertension allowing the discrimination between mechanical pressure effects and Angiotensin II’s pharmacological action. We utilized an advanced bioreactor system capable of producing adjustable flow rates to culture human umbilical vein endothelial cells (HUVEC). This system allows for the investigation of the possible effects of Angiotensin II and/or an increase in intraluminal pressure (via the Live-Pa pressure-actuation device) exerted directly upon the HUVEC monolayer without simulating transmural pressure. Key hypertension-associated inflammatory markers, such as NF-kB, p38MAPK, Interleukins (IL)-6/8, and Endothelin-1 (ET-1), were subsequently assessed. Angiotensin II induced HUVEC NF-kB and p38MAPK phosphorylation, and elevated IL-6 and ET-1 secretion, with a trend in IL-8 increase. Live-Pa alone enhanced NF-kB and p38MAPK and influenced cytokine/chemokine secretion. Combined stimuli significantly augmented the inflammatory parameters as compared to unstimulated cells, suggesting a synergistic effect between chemical and mechanical stimuli. Overall, these *in vitro* results demonstrate both key consistencies (e.g., NF-kB and p38MAPK activation) and specific distinctions (e.g., no significant IL-6 increase in Live-Pa-exposed versus control HUVEC) when compared to published data from hypertensive versus normotensive animal models. The proposed advanced *in vitro* model may successfully reproduce some features of vascular function in hypertension and simulate hemodynamic conditions by controlled flow with adjustable pressure parameters. Crucially, this system allows discrimination between mechanical blood pressure effects and Angiotensin II’s pharmacological action on the endothelium, paving the way for understanding pathophysiological mechanisms and developing new therapies. Established methods make it possible that studies on cultured endothelial cells will be better comparable to the results of *in vivo* studies, thus directly supporting the 3Rs framework—Replacement, Reduction, and Refinement—which is essential for high-standard and ethical research.

## 1 Introduction

Hypertension stands as one the most significant preventable risk factor for cardiovascular disease, which is the leading cause of mortality worldwide. This chronic condition affects several hundred millions of people globally and often develops progressively over years before manifesting serious health complications (Danaei et al., 2009; Zhou et al., 2017). One of the main challenges is to understand the intricate network of biochemical connections underlying the pathophysiological mechanisms responsible for this risky condition. Therefore, targeted investigation are required to unmask key functional mechanisms and pathways behind hypertension. In the last decade, extensive studies have confirmed what had been increasingly evident: inflammation is an essential player in the development and progression of the hypertensive condition. Animal models and clinical studies have established connections between hypertension and activation of both innate and adaptive immune systems, which can cause systemic damage (Shi et al., 2010; Singh et al., 2014; Tracey, 2014; Santisteban et al., 2015; Calvillo et al., 2019; Youwakim and Girouard, 2021; Pugh and Dhaun, 2021; Hengel et al., 2022; Xu et al., 2023; Aboukhater et al., 2023; Guzik et al., 2024; Nguyen et al., 2024; Zubcevic et al., 2014; Carnevale et al., 2014; Harrison, 2014). *In vitro* and *in vivo* experimental research must investigate the interconnected pathways that regulate pressure responses, as disruptions in these networks can lead to various disorders, including abnormal blood pressure levels (Calvillo et al., 2019; Zubcevic et al., 2014; Kapoor et al., 2016).

With such a complex network to study, basic research faces methodological challenges: animal models provide comprehensive but overly complex systems with multiple interacting variables, while traditional *in vitro* cell cultures are too simplistic, lacking physiological conditions and intercellular communication. Despite limitations in isolating specific pathways, animal experiments still dominate hypertension research (Cook et al., 2001; Healey et al., 2003; Mitchell et al., 2007; Cook and Re, 2012; Lerman et al., 2019); therefore, advanced *in vitro* systems are needed to bridge the gap between simple cell cultures and animal experiments, while better replicating physiological conditions without multiple organ system interference (Vozzi et al., 2011; Ucciferri et al., 2014; Marchesi et al., 2020). These tools align with the 3Rs framework (Replacement, Reduction, Refinement) (MacArthur, 2018).

Some *in vitro* dynamic models have been developed to mimic at least to some degree the *in vivo* endothelial environment. The fundamental principles for constructing adjustable shear stress flow chambers, critical for generating the flow regimes, have recently been established (Fallon et al., 2022). The use of human umbilical vein endothelial cell (HUVEC) monolayers subjected to pulsatile perfusion to construct simplified vascular models in bioreactors is a recognized approach in vascular tissue engineering (Helms et al., 2021). Furthermore, the influence of shear rate on inflammatory responses, such as adhesion molecule expression, in microfluidic *in vitro* models has been demonstrated (Rotenberg et al., 2012), with prior work also examining the combined effects of pulsatile wall shear stress and tensile hoop strain in HUVEC (Breen et al., 2010).

The current study aimed to develop an advanced bioreactor dynamic model connectible to a peristaltic pump which reproduces mechanical flow-dependent shear stress, and to test the Live-Pa device able to increase the intraluminal pressure directly on the HUVEC monolayer. Such model mirrors physiological conditions that the endothelium, a key initiator and sensor of hemodynamic forces, experiences in living organs (Vozzi et al., 2011; Vernazza et al., 2021). Furthermore, this system should allow the controlled exposure of the cells to soluble mediators, such as Angiotensin II (ANG II), a peptide hormone causing vasoconstriction, blood pressure increase and direct chronic inflammatory effects on vascular cells, thereby enabling the distinction between mechanical blood pressure effects and pharmacological actions. This setup might favour the understanding of the mechanisms leading to hypertension and developing of new therapies.

Several inflammatory factors were published in the literature as significantly associated with hypertensive condition. Among them, Nuclear factor kappa-light-chain-enhancer of activated B cells (NF-kB), p38 Mitogen-activated protein kinases (p38 MAPK), Interleukin-8 (IL-8) and Interleukin-6 (IL-6), were found to be overexpressed in animal model of hypertension and increased in serum of hypertensive patients (Calvillo et al., 2019; Ju et al., 2003; Dai et al., 2016; Liu et al., 2017; Tanase et al., 2019; Kim et al., 2020; Mohammed et al., 2022; Song et al., 2022; Lee et al., 2006; Basson et al., 2015; Geng et al., 2019). Accordingly, we investigated these inflammatory mediators in our model. We also evaluated Endothelin-1 (ET-1) as a potent vasoconstrictor peptide, focusing on its critical role as a major product of vascular endothelial cells, which is known to be increased in several experimental models of hypertension (Mohammed et al., 2022; Zhang et al., 2023; Xue et al., 2022; Schiffrin, 1999).

## 2 Materials and methods

### 2.1 Modular dynamic multi-compartmental cell culture system

The modular dynamic device, developed by IVtech (IVTech Srl, Ospedaletto, PI, Italy), allows 2D or 3D cell culturing both in static and dynamic conditions. This system provides two different transparent 24 well plate-like culture chambers (bioreactors): Live-Box (LB) type 1 and type 2. LB1 is equipped with a removable glass slide supporting cell monolayer or 3D constructs and a flow inlet and outlet for the perfusion of culture media. LB2 allows the modelling of physiological barriers *in vitro* through a porous selective membrane (specific for each cell type) housed in a removable holder. It also has two flow inlets and outlets. In our study, we employed only LB1 for culturing endothelial cells (ECs) in 2D conditions.

**FIGURE 1:**
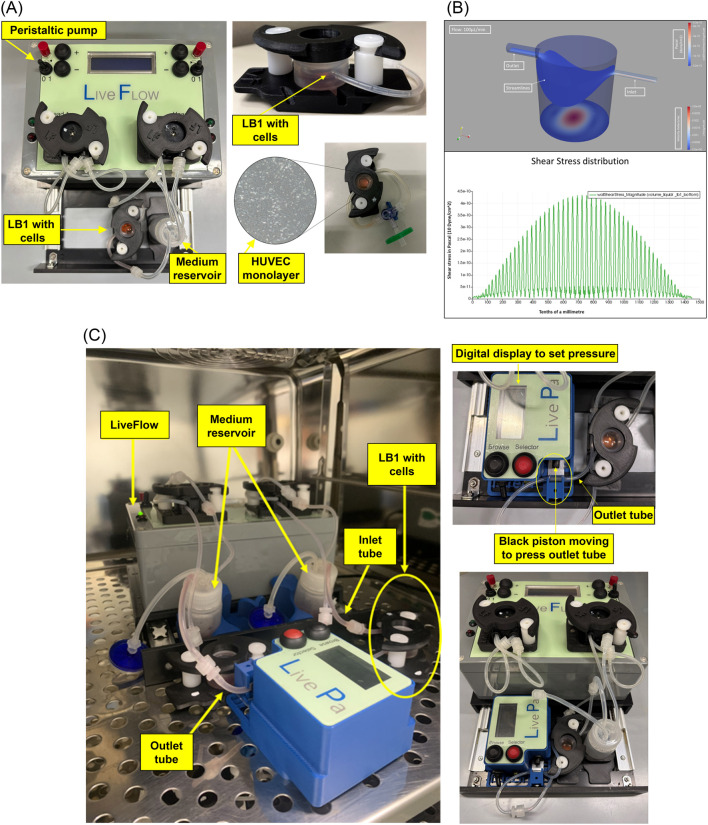
Setting. Dynamic *in-vitro* culture system. Original photos of the equipment and colorimetric map of the shear stress distribution: **(A)**- the peristaltic pump (Live Flow) able to perform an adjustable flow rate (100–450 μl/min) is connected both to bioreactor and reservoir mimicking blood flow circulation (left). Details of the LB1 (right, up) and magnification of the HUVEC monolayer inside the LB1 (right, bottom) are shown. **(B)**- colorimetric map of the shear stress distribution with wall shear stress magnitude diagrams. Up: the map illustrates the distribution of shear stress across the surface of the slide inside the LB1. The velocity is also indicated along the streamlines. Bottom: Shear stress distribution. The graph illustrates the shear stress distribution across the diameter of the LB1 slide, measured in the direction perpendicular to the inlet-outlet axis. The x-axis represents the slide’s length in tenths of a millimetre, and the y-axis shows the shear stress measured in Pascals (1 Pa = 10 dyne/cm^2^). **(C)** - Dynamic device with Live Pa (left); the LB1connected to Live-Pa with the black piston inducing pressure increase (center); and the dynamic device equipped with two LB1 and Live-Pa inside the incubator (right).

A peristaltic pump (Live-Flow, IVTech) connected to bioreactors and reservoirs applies an adjustable flow rate (range 100–450 μL/min) mimicking blood flow circulation (Figure 1A). The current model primarily addresses the effects of flow (shear stress) and pressure exerted directly upon the monolayer. Currently, the model applies a horizontal flow, whose pressure acts on the monolayer without crossing it (i.e., not mimicking transmural pressure). The immediate focus of this model is to consider the blood vessel as a simple conduit, without examining phenomena of extravasation. The shear stress ( τ ) applied to the endothelial monolayer under the flow conditions described in the study is 6x10^-10^ pascal (Pa). The general formula used for calculating shear stress τ is:

$$\tau =-µ\;x\;\left(dv/dz\right)$$

Where µ is the dynamic viscosity of the fluid and dv/dz represents the velocity gradient across the channel’s cross-section.

This formula, accounting for the specific geometry and flow profile, was calculated automatically by the numerical simulation software, Simflow, used to generate the colorimetric map of the shear stress distribution (Figure 1B).

The device may be implemented by a pressure modulator, Live-Pa, which allows to increase the hydrodynamic pressure with a motorized piston reducing the lumen of the outlet tube of a chamber and increasing the pressure like a disease scenario (e.g., hypertension) (Figure 1C).

The Live-Pa applies pressure to cells in the bioreactor subjected to flow, which can be varied from 101.3 to 151.1 to 202.6 Pascal, corresponding to 0.75, 1.13, and 1.52 mmHg, respectively. Live-Pa offers the capability to increase the pressure incrementally by 50%, 100%, or 200%. A 50% increase was selected in our study to better model human hypertension, where such an increase is often compatible with the clinical condition. The crucial parameter is the ratio between the applied overpressure and the basal pressure of the system, rather than the absolute pressure value itself. This model is scaled in relation to the physiological *in vivo* reality; therefore, there is no direct 1:1 correspondence between the specific values used in the model and the actual values measured *in vivo*, as well as there isn't a direct 1:1 mapping between the model’s biological structures and those found *in vivo*. The change in velocity is localized to the segment of the tubing undergoing Live-Pa compression downstream the bioreactor. As we were operating within a closed-loop system, the peristaltic pump imposed a constant flow, overriding any transient, localized velocity changes that might occur due to compression in a different part of the circuit.

Endothelial monolayer in LB1 is shown in Figure (Figure 1A).

### 2.2 Cells

Human umbilical vein endothelial cells (HUVEC) were purchased from Promocell (Heidelberg, Germany) and grown in the provided Endothelial Cell Growth Medium Ready-to-use (Promocell) in humidified incubator at 37 °C and 5% CO_2_. Confluent cells were detached with specific Detachkit-30 (Promocell), and 80.000 cells in 2 mL of complete medium were seeded in 24 well/plate and 200,000 cells in 20 µL in LB1. These cell numbers were used in all the investigated conditions to avoid any experimental variability.

Four hrs later, 2 mL of complete medium were added in LB1 and cells were cultured in static state for further 24 h to reach the confluence.

Parallel experiments were performed in the presence of 100 μL/min of flow for further 24 h to mimic dynamic environment.

Confluent HUVEC cells were subjected to two distinct stimuli, applied individually or in combination. The pharmacological stimulus consisted of ANG II at a concentration of 1,000 nM (Sigma-Aldrich, St Louis, MO, United States, catalog number A9525), which was applied for a total duration of 24 h under both static and dynamic flow conditions. The ANG II dose and exposure regimen were determined based on relevant literature (Marchesi et al., 2020; Deng et al., 2015), evaluating the effect of the molecule on the expression of several immune factors in HUVECs and other cardiovascular cells. The mechanical stimulus by Live-Pa device, applied an acute 50% pressure increase to the circuit. This mechanical stress was maintained for a specific duration of 2 h, either alone or combined with ANG II.

Cells were incubated with LPS (1 μg/mL, Sigma-Aldrich, catalog number L4516) or medium alone for 24 h in the conditions described above, as experimental controls.

HUVEC morphology and uniform monolayer preservation were evaluated by inverted microscopy (Olympus CKX53, EP50, Shinyuku, Tokio, Japan) in all the experimental settings (Figure 1A).

**FIGURE 2:**
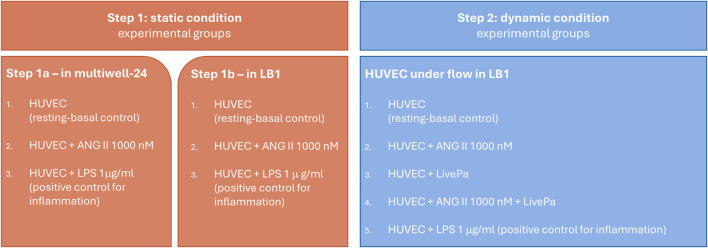
Experimental Design. Human vein umbilical endothelial cell (HUVEC) cultures in static (step 1) and dynamic conditions (step 2). Confluent cells were treated with ANG II for 24 h in both conditions. In step 2, 50% pressure increase was applied by Live-Pa to the circuit for 2 h, alone or in combination with ANG II.

The detailed experimental design is shown in Figure 2.

### 2.3 Evaluation of NF-kB and p38MAPK activation

The activation (phosphorylation) rate of NF-κB) and p38MAPK was assessed in HUVEC seeded in LB1 incubated with ANG II (1,000 nM) for 24 h both in static and dynamic conditions, in the presence or absence of Live-Pa (2 h) by Western blotting. Control cultures were treated with LPS (1 μg/mL) or medium alone.

Total proteins were isolated using RIPA Lysis Buffer (Cell Signaling Technology, Danvers, MA, United States, catalog number BK9806S) added with 1% Protease and Phosphatase Inhibitor Cocktail (Sigma-Aldrich, catalog number P8340) and protein concentration was measured using the BCA Protein Assay Kit (ThermoFisher Scientific, catalog number 23227).

Proteins (10 μg/lane) along with a molecular weight ladder (ThermoFisher Scientific, catalog number #SM1841) were fractionated by NuPAGE BIS-TRIS by 4%–12% SDS-polyacrylamide pre-cast gel electrophoresis (ThermoFisher Scientific, catalog number NP0321BOX) and transferred to nitrocellulose using iBlot 3 Transfer Stacks Nitrocellulose (ThermoFisher Scientific, catalog number IB33002).

The membranes were blocked for 2 h at room temperature (RT) in PBS/0.05% Tween 20 (PT) (Cell Signaling Technology) containing 5% non-fat dry milk (Santa Cruz Biotechnology, Dallas, TX, United States) and incubated overnight at 4 °C with anti-human NFκB (1:1,000 in PT plus 5% BSA, Cell Signaling Technology, catalog number #8242) or anti-human phosphorylated NFκB (pNFκB, 1:1,000 in PT plus 5% BSA, Cell Signaling Technology, catalog number #3039) or anti-human p38MAPK (1:1,000 in PT plus 5% BSA, Cell Signaling Technology, catalog number #9212) or anti-human phosphorylated p38MAPK (pp38MAPK, 1:1,000 in PT plus 5% BSA, Cell Signaling Technology, catalog number #9211) After three washes, the membranes were incubated for 1 h at RT in PT/5% non-fat dry milk plus HRP-conjugated secondary antibodies (1:5000, Cell Signaling Technology, catalog number #7074) and developed using ECL (Westar Supernova, Cyanagen, Bologna, IT, catalog number XLS3,0100). Signals were detected using radiographic films (Kodak, Rochester, NY, United States). The ImageJ software (LI-COR Biosciences, Lincoln, NE, US) was used to analyze and quantify gels. Results were expressed as the ratio of phosphorylated to non-phosphorylated forms, normalized to the respective control.

To identify the protein of interest when the exposure time was too short to impress the X-ray film, its position was identified by a protein ladder transferred onto nitrocellulose membrane from the SDS-polyacrylamide pre-cast gel (Liang et al., 2013) (Supplementary Figure S1).

### 2.4 Measurement of inflammatory mediators secretion

Supernatants were collected at the end of each experiment and frozen at −20 °C for further analyses. The selected mediators IL-6 and IL-8 were measured using the automated microfluidic analyzer ELLA (Bio-Techne, Minneapolis, MN, United States, Simple plex Cartridge kit, catalog number 356322). ELLA-SimplePlex technology allows biomarkers to be quantified in 25 µL of supernatants in a multiplex format, with extremely high reproducibility. This system uses cartridges pre-loaded with everything necessary for biomarker quantification, including the calibration curve. To perform the assay, the sample was diluted in the provided buffer and loaded into the cartridge, for ELLA analysis. The concentrations of each analyte (pg/ml, mean of three reading) were obtained from the specific calibration curve using the system’s software.

Minimum detectable level for IL-6 is 0.7 pg/mL (range 0.7–2.652 pg/mL) and for IL-8 is 0.08 pg/mL (range 0.19–1.8 pg/mL). ET-1 was quantified by commercial Kit ELISA (Bio-Techne, catalog number DET100), according to the manufacturer’s instructions. A standard curve was generated by plotting the mean absorbance for each standard on the y-axis against the concentration on the x-axis. The data were linearized by plotting the log of the ET-1 concentration versus the log of the optical density (OD). The sample concentration was calculated interpolating OD values in the standard curve ranging from 0 pg/mL to 25 pg/mL.

The minimum detectable dose of ET-1 is 0.031(range 0.031–0.207 pg/mL).

The sample concentration obtained from the two different systems was multiplied by a factor 7 corresponding to the total volume circulating in the device.

### 2.5 Statistical analyses

Continuous variables were shown as mean and standard error (SEM). The Mann-Whitney test was used for comparisons of quantitative variables between two groups. Due to the exploratory nature of the study, no comparison of multiple tests was applied. All the analyses were performed with GraphPad Prism 5.01. Statistical significance was set at the 0.05 level. All P-values were two-sided.

## 3 Results

### 3.1 Intracellular signaling pathways

**FIGURE 3:**
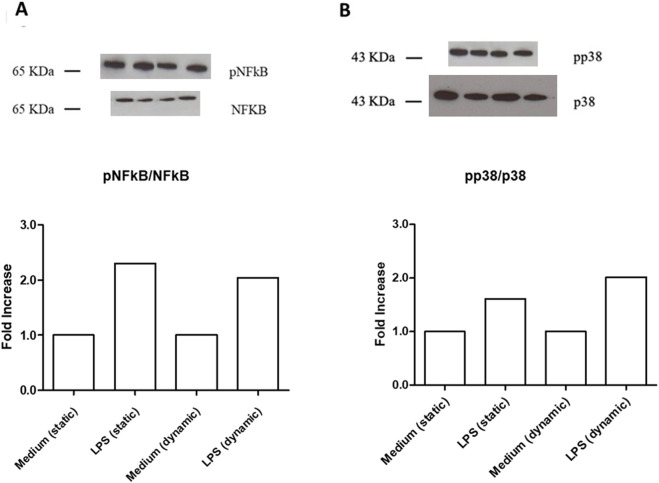
Intra-cellular signaling pathways in unstimulated or LPS-stimulated HUVEC seeded in LB1, cultured in static or dynamic conditions. NFkB **(A)** and p38MAPK **(B)** phosphorylation after stimulation with LPS (1 μg/mL). Endothelial cells seeded in LB1 for 24 h were incubated with LPS or medium alone both in static and under flow for further 24 h, as experimental control. Results are expressed as the ratio of phosphorylated to non-phosphorylated form, normalized to the respective control and evaluated using Image J software. Western Blotting images are representative of a single experiment. Histograms represent mean of three independent experiments. pNFkB: phosphorylated NFkB; p38MAPK: phosphorylated p38MAPK.

LPS (1 μg/mL) strongly activated both NFkB and p38 MAPK phosphorylation in HUVEC seeded in LB1, under static and dynamic conditions, compared to the culture medium alone (Figures 3A,B). These observations confirm the functional validation and biological responsiveness of the device, as they are consistent with those obtained using traditional *in vitro* plates (Wang et al., 2023; Raschi et al., 2003; Cai et al., 2021), specifically demonstrating that our system accurately captures the LPS-induced activation of key inflammatory signaling pathways.

**FIGURE 4:**
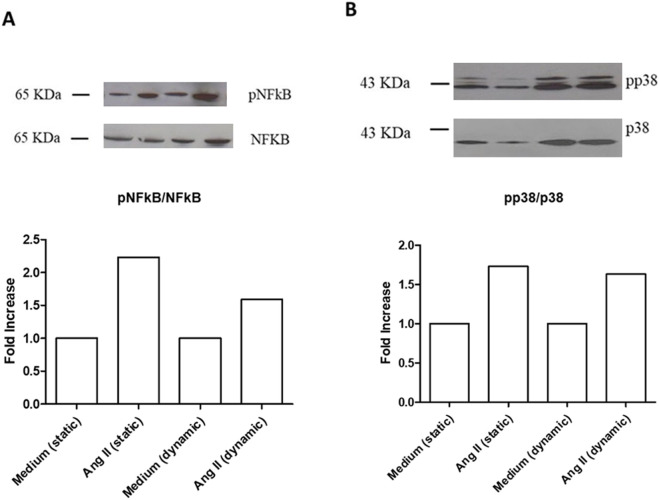
Intra-cellular signaling pathways in unstimulated or Angiotensin II-stimulated HUVEC seeded in LB1, cultured in static or dynamic conditions. NFkB **(A)** and p38MAPK **(B)** phosphorylation after stimulation with ANG II (1,000 nM). Endothelial cells seeded in LB1 for 24 h were incubated with Angiotensin II or medium alone both in static and under flow for further 24 h. Results are expressed as the ratio of phosphorylated to non-phosphorylated form, normalized to the respective control and evaluated using Image J software. Western Blotting images are representative of a single experiment. Histograms represent mean of three independent experiments. pNFkB: phosphorylated NFkB; p38MAPK: phosphorylated p38MAPK.

**FIGURE 5:**
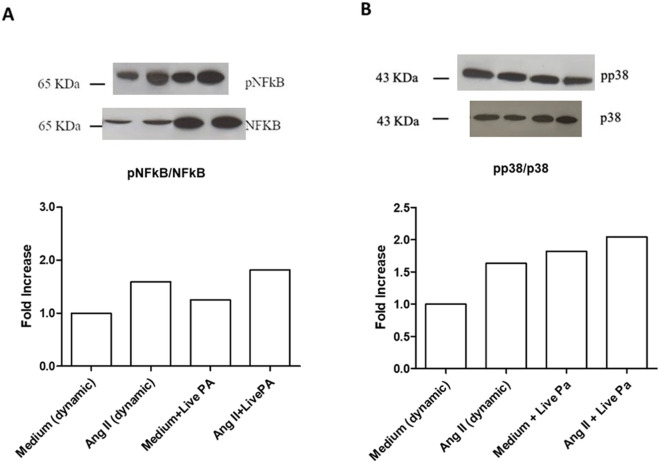
Intra-cellular signaling pathways in HUVEC seeded in LB1, cultured in static or dynamic conditions, unstimulated or stimulated with Angiotensin II and Live-PA, individually or in combination. NFkB **(A)** and p38MAPK **(B)** phosphorylation after stimulation with ANG II (1,000 nM) alone or in combination with Live-Pa. Endothelial cells seeded in LB1 for 24 h were stimulated with ANG II under flow for 24 h. Live-Pa was applied for further 2 h in the presence of medium or ANG II. Results are expressed as the ratio of phosphorylated to non-phosphorylated form, normalized to the dynamic control and evaluated using Image J software. Western Blotting images are representative of a single experiment. Histograms represent mean of three independent experiments. pNFkB: phosphorylated NFkB; p38MAPK: phosphorylated p38MAPK.

Treatment with ANG II (1,000 nM) for 24 h induced high phosphorylation of NFkB in static conditions and under flow (Figure 4A). The same effect was observed for p38 MAPK phosphorylation in both dynamic and static milieu (Figure 4B). When Live-Pa was applied to the circuit for 2 h, the activation rate of the intracellular signaling pathways (NFkB and p38 MAPK) was upregulated with respect to the medium alone (Figures 5A,B). No differences were observed in NFkB phosphorylation levels between the ANG II/Live-Pa combination and ANG II alone, while an increase in p38 MAPK activation was detected in the cells treated with ANG II in combination with Live-Pa versus ANG II alone (Figures 5A,B).

### 3.2 Cytokine and chemokine secretion

**FIGURE 6:**
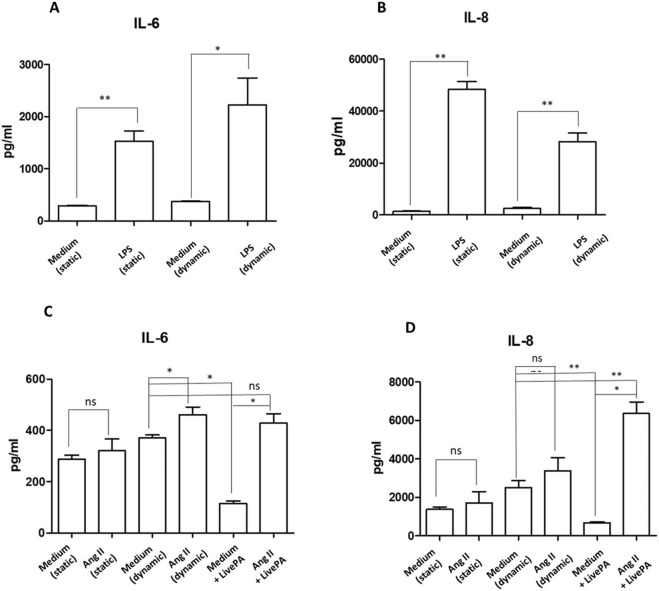
Cytokine secretion. IL-6 **(A)** and IL-8 **(B)** protein levels in supernatants from HUVEC resting or stimulated with LPS (1 μg/mL) both in static and under flow for 24 h, as positive experimental control. Histograms represent mean +standard error of the mean (SEM). *p < 0.05 **p < 0.01 versus respective controls (static or dynamic medium). IL-6 **(C)** and IL-8 **(D)** protein levels in supernatants from HUVEC resting or stimulated with ANG II (1,000 nM) and Live-Pa, individually or in combination. Endothelial cells seeded in LB1 for 24 h were stimulated with ANG II (1,000 nM) under flow for 24 h. Live-Pa was applied for further 2 h in the presence of medium or ANG II. (Histograms represent mean +standard error of the mean (SEM) *p < 0.05 vs. medium + Live-Pa. **p < 0.01 versus medium (dynamic).

Endothelial cells treated with LPS (1 μg/mL) for 24 h resulted in a significant increase of the cytokine IL-6 secretion both in static and dynamic conditions (Figure 6A). Similarly, IL-8 levels were significantly upregulated in LPS treated cells in both conditions (Figure 6B). These findings are consistent with results observed when endothelial cells are cultured and stimulated with LPS in traditional plates, confirming the validity of the multicompartmental system as an advanced culture model (Wang et al., 2023; Raschi et al., 2003; Cai et al., 2021; Li et al., 2006).

Exposure to ANG II (1,000 nM) for 24 h led to a slight increase in IL-6 levels in the supernatants collected from LB1 under static conditions. When flow was applied, cytokine levels were significantly up-regulated. Moreover, mechanical stimulation with Live-Pa for 2 h in combination with ANG II significantly enhanced IL-6 secretion compared to Live-Pa alone, but induced a slight increase versus control (medium) (Figure 6C).

Treatment with ANG II for 24 h caused a mild increase in chemokine IL-8 secretion both in static and dynamic environments. Conversely, when the Live-Pa device was applied in combination with ANG II treatment, significantly higher IL-8 levels were observed (Figure 6D).

### 3.3 Endothelin I release

**FIGURE 7:**
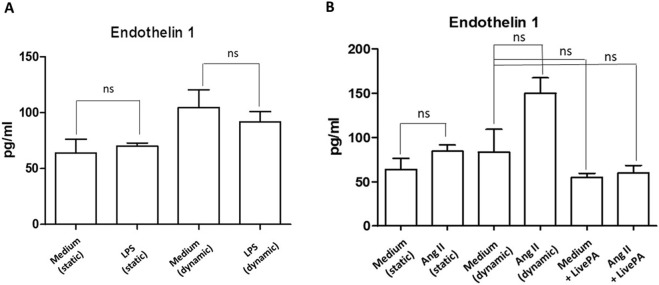
Endothelin I secretion. **(A)** Endothelin I secretion in HUVEC seeded in LB1, cultured in static or dynamic conditions, and unstimulated or stimulated with LPS (1 μg/mL), as positive experimental control. Histograms represent mean +standard error of the mean (SEM). **(B)** Endothelin I secretion in HUVEC seeded in LB1, cultured in static or dynamic conditions, resting or in the presence of ANG II (1,000 nM) and Live-Pa, individually or in combination. Endothelial cells seeded in LB1 for 24 h were stimulated with ANG II (1,000 nM) under flow for 24 h. Live-Pa was applied for further 2 h in the presence of medium or ANG II. Histograms represent mean +standard error of the mean (SEM).

When treated with LPS (1 μg/mL), in both static and dynamic conditions, Endothelin I levels did not change (Figure 7A). Treatment with ANG II (1,000 nM) induced a more evident upregulation of the investigated protein in the dynamic environment compared to the static one, even if it was not statistically significant. When Live-Pa was added to the circuit, a downregulation of Endothelin I levels was observed, compared to ANG II alone (Figure 7B).

## 4 Discussion

This study reports a new advanced *in vitro* approach utilizing a millifluidic platform to reproduce and investigate the early signaling features of hypertensive stress. The specific focus is on the individual and combined effects of mechanical stress and ANG II on endothelial NF-kB and p38 MAPK activation, and associated inflammatory cytokine release (IL-6 and IL-8). To investigate the early molecular signaling events and acute activation of the two pathways triggered by the combined hemodynamic and biochemical stress, we intentionally applied a short 2-h mechanical stimulus. Such acute stress should highlight the initial endothelial response to hypertensive stress in our system.

The critical innovation lies in incorporating controlled flow with adjustable pressure parameters, which enables simulation of the hemodynamic conditions seen in hypertensive states and reproduces certain features of inflammation, a recognized hallmark of the condition. Accordingly, over the past two decades, research has increasingly revealed a clear connection between inflammation and hypertension, with evidence from both human patients and animal studies demonstrating that inflammation plays several crucial roles in how hypertension develops and progresses (Calvillo et al., 2019; Ju et al., 2003; Dai et al., 2016; Basson et al., 2015; Park et al., 2007; Bao et al., 2007; Luft, 2001; Ruiz-Ortega et al., 1998; Dornas et al., 2017; Luo et al., 2015; Miguel-Carrasco et al., 2010; Hammer et al., 2017; Winklewski et al., 2015; Benigni et al., 2010; Harrison et al., 2011; Marvar et al., 2011; Kim et al., 2015; Martynowicz et al., 2014; Zhang et al., 2022; Patrick et al., 2021; Kim et al., 2011).

Based on data in the literature, NF-kB, p38MAPK, IL-8 and IL-6 appear significantly associated with hypertensive condition. Published studies strongly highlights the pronounced difference in inflammatory cytokine levels as well as in pro-inflammatory transcription factors and signalling pathways between spontaneously hypertensive and normotensive rats, further confirming the critical role of inflammation in the pathophysiology of hypertension. In details, NF-κB, a key intracellular inflammatory signal protein whose expression is elevated in arterial vascular cells during hypertension, regulates oxidative stress, angiogenesis, and apoptosis, while p38 MAPK controls cell differentiation and apoptosis. Both pathways are increased in hypertensive rats’ vessels respect to normotensive control animals (Calvillo et al., 2019; Ju et al., 2003; Dai et al., 2016; Liu et al., 2017; Basson et al., 2015; Duansak and Schmid-Schönbein, 2013). The IL-8 chemokine and the pro-inflammatory cytokine IL-6 are overexpressed in hypertension, with IL-6 promoting ANG II-induced hypertension (Calvillo et al., 2019; Kim et al., 2020; Mohammed et al., 2022; Song et al., 2022; Lee et al., 2006; Geng et al., 2019; Kim et al., 2015; Martynowicz et al., 2014; Kim et al., 2011; Dusi et al., 2016).

In our study, these markers were measured in HUVEC subjected to the consequences of mechanical blood pressure increase and the pharmacological action of ANG II. The two different stimuli were applied individually or in combination, in order to discriminate between a mechanical pressure-increase impact and the biochemical effects of ANG II. NF-kB and p38-MAPK activation increased, particularly after Live-Pa/ANG II combined exposure, confirming their *in vitro* roles as players in the hypertensive state. This effect was consistent with the trends observed in vessels from SHR vs. normotensive Wistar Kyoto rats (WKY) in *in-vivo* studies (Duansak and Schmid-Schönbein, 2013; Bhatt et al., 2014; Luo et al., 2021; Chen et al., 2015; Hanson et al., 2016; Zhang et al., 2019). Similarly, IL-8 secretion after hypertensive stimuli had more than doubled after the combined treatment, compared to control medium. These results mirror observations from basal measurements in hypertensive animal vessels compared to the WKY ones (Kim et al., 2015; Kim et al., 2011), and suggest that our millifluidic model successfully captures key early activation of signaling pathways characteristic of hypertensive stimuli in the vasculature. In our *in vitro* model IL-6 showed only a slight (10%) increase after HUVEC exposure to Live-Pa/ANG II combined treatment compared to control cultures. These results evidence a key difference from *in-vivo* observations in hypertensive animals, in which circulating IL-6 is higher when compared to normotensive rats (Geng et al., 2019; Li et al., 2011). This discrepancy likely stems from the absence of paracrine amplification; in fact, high IL-6 levels in *in-vivo* hypertension have been shown to be mediated by the infiltration and activation of immune cells, such as T lymphocytes, which are the main source of the massive systemic IL-6 increase and are missing in our 2D monoculture (Senchenkova et al., 2019).

When mechanical and biochemical stimuli were applied separately, an interesting observation emerged: the secretion of inflammatory cytokines IL-8 and IL-6 under stimulation with Live-Pa alone was significantly lower than in the control flow condition. While our model is observational and cannot provide definitive mechanistic insight, the transient nature of the mechanical stimulus bears a certain resemblance to the physiological blood pressure elevation experienced during physical exercise. The resulting increased oxygen demand stimulates the heart to increase its rate, with a physiological rise in blood pressure as a normal adaptation to physical exertion. Data in literature suggesting an association between exercise and anti-inflammatory effects may offer a plausible explanation, though unproven, for the observed reduction in cytokine secretion under Live-Pa alone (Briones and Touyz, 2009; Dorneles et al., 2016). On the other hand, ANG II treatment alone caused significant inflammatory activation, evidenced by both significantly increased phosphorylation of NF-κB and p38-MAPK at the cellular level and enhanced IL-8 content, which is consistent with literature findings (Guo et al., 2006; Nabah et al., 2004) and supports the efficiency of our model.

When we investigated ET-1, a potent vasoconstrictor peptide, the molecule showed an endothelin-independent pattern after exposure of HUVEC to Live-Pa + ANG II, similarly to what has been described in SHR animals (Schiffrin, 1999).

The ET-1 behaviour observed in our model is highly significant, as the endothelin axis is centrally involved in the vascular pathology of hypertension. Our *in vitro* platform might provide mechanistic insight into how isolated or combined hemodynamic and biochemical stimuli modulate this potent vasoconstrictor at the level of the endothelium. This early mechanism is crucial, given that ET-1 and its related pathways are known to drive cardiac and vascular remodeling in hypertension, representing a potential target for novel therapeutic strategies (Gaydarski et al., 2025). In a broader translational context, by specifically examining ET-1 and other inflammatory markers, the output of our system can potentially contribute to the understanding of early biochemical steps that lead to systemic consequences of hypertension, such as organ-specific remodeling and functional deterioration observed in key targets (e.g., the kidney) (Gaydarski et al., 2024).

Collectively, our findings point toward a synergistic pro-inflammatory effect between chemical and mechanical stimuli on endothelial cells, and support the potential of our dynamic multi-compartmental cell culture system to model specific, early signaling features of *in-vivo* hypertensive stress.

Moreover, allowing the identification of potential critical mechanisms at endothelium level, our advanced dynamic platform might offer a valuable screening tool for investigating known and newly developed anti-hypertensive molecules.

## 5 Future perspectives

We are going to test potential protective effects of a consolidated privileged scaffolds in medicinal chemistry (core structures of bioactive compounds), as pyrazole, imidazopyrazole derivatives (Meta et al., 2017; Selvatici et al., 2013; Lusardi et al., 2022) and others (Alfei et al., 2022). These molecules were previously identified as inhibitors or modulator of hypertension-related inflammatory factors, as p38MAPK, NFkB signalling and cytokine production.

From a pharmacological perspective, our dynamic system with Live-Pa could serve as a platform for developing and characterizing new therapeutic molecules for hypertension-related inflammation. This advanced dynamic *in vitro* model could reduce animal use and costs while providing an alternative method for detecting adverse effects in initial pharmacological screening. Future applications will also include a more complex multi-compartmental dynamic model in which cells from different organs and systems, including the immune system, may interact (Bodio et al., 2024).

## 6 Study limitations

We took into account other factors known to play a role in hypertension, i.e. IL-17, (Calvillo et al., 2019; Harrison, 2014; Robles-Vera et al., 2021; Guo et al., 2015; Madhur et al., 2010), VEGF (Advani et al., 2007; Wang et al., 2018) and CXCL12 (Calvillo et al., 2019; Liu et al., 2018; Masson et al., 2015). However, these mediators were undetectable by ELISA in our experimental conditions (data not shown), possibly owing to their low secretion levels and/or high dilution in the flow circuit. To overcome this bias, we might concentrate the culture supernatants and/or employ high sensitivity assays. A further limitation concerns the use of HUVEC, which do not necessarily represent the microcirculation, which undoubtedly plays a relevant role in hypertension development as well as in the macrocirculation (Laurent et al., 2022). Finally, our study was purposefully designed to be purely exploratory and hypothesis-generating, rather than a primary, confirmatory hypothesis test. Our proof-of-concept analysis was focused on evaluating the performance of the platform and its efficiency in mimicking *in vivo* hypertensive conditions. In this context, choosing not to apply a multi-group correction is a recognized methodological approach.

Future studies are being planned to confirm these results and extend the research to different vascular cells (i.e. microcirculation endothelial cells, smooth muscle cells), also implementing the complexity of our multifluidic system.

## Data Availability

The datasets generated for this study can be found in the ZENODO repository [DOI 10.5281/zenodo.17191714].

## Supplementary material

SUPPLEMENTARY FIGURE S1 Image of uncropped blots.

SUPPLEMENTARY TABLE S1 Excel file with reference details of studies reporting the baseline differences in the levels of inflammatory markers NF-kB, p38MAPK, Interleukins (IL)-6/8, between SHR and WKY blood vessels.
