# Supplementary figures and images for: Direct simulation of hypertensive stress on endothelial cells: a streamlined model of in-vitro-hypertension
Source: Front Physiol. 2026 Jan 14;16:1724932. doi: 10.3389/fphys.2025.1724932 (PMC12847034; doi:10.3389/fphys.2025.1724932)

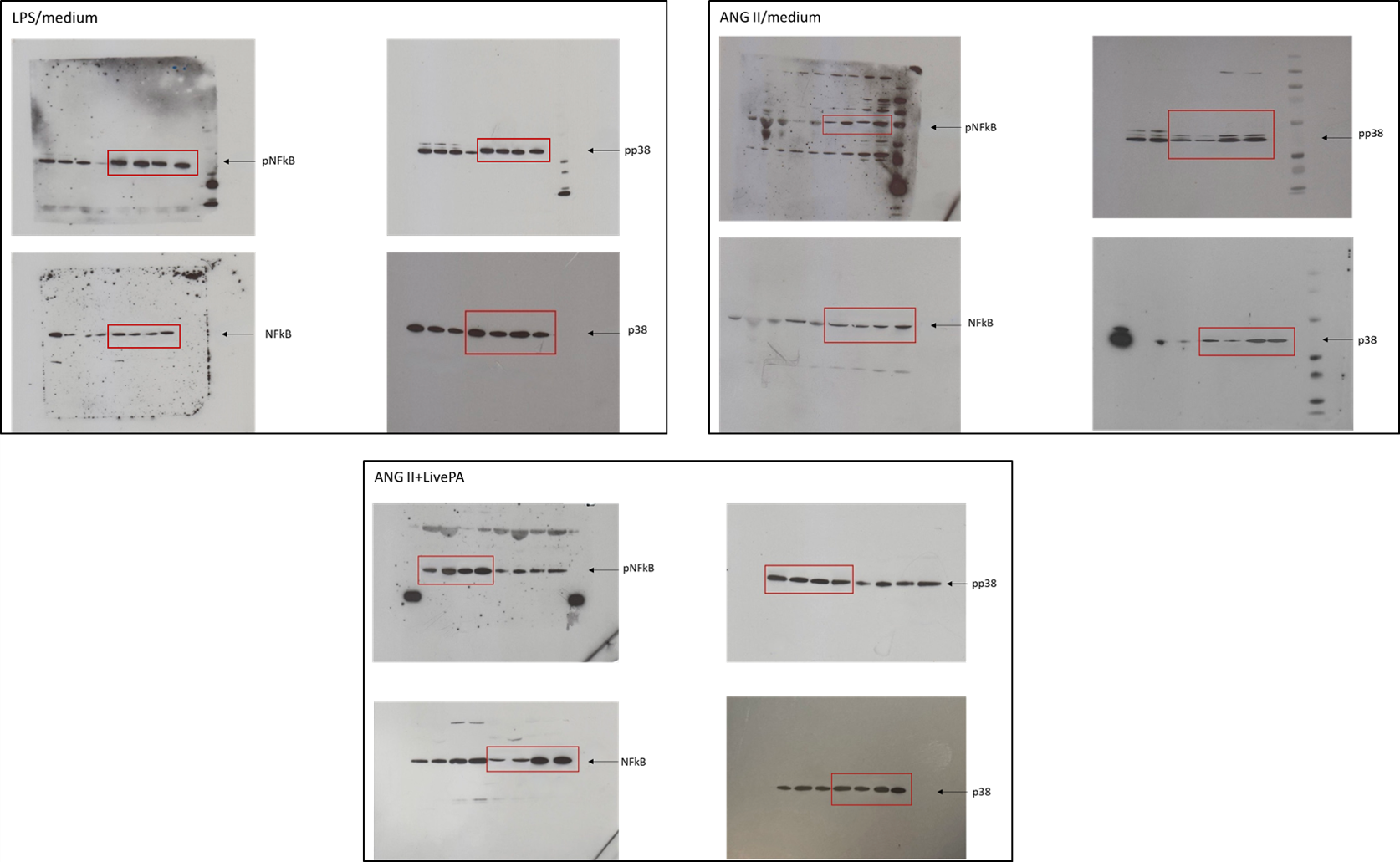

Supplement: Supplementary file 1 [file Image1.tif]
